# Integrating Health Care to Meet the Needs of the Mother–Infant Pair: A Call for Papers for Year 3 of the Maternal Health Task Force–PLOS Collection

**DOI:** 10.1371/journal.pmed.1001559

**Published:** 2013-11-26

**Authors:** Kate Mitchell, Mary Nell Wegner, Ana Langer, the PLOS Medicine Editors

**Affiliations:** Harvard School of Public Health, Women and Health Initiative, Maternal Health Task Force, Boston, Massachusetts, United States of America

## Abstract

The Maternal Health Task Force (MHTF) and *PLOS Medicine* issue the call for papers for Year 3 of the MHTF-PLOS Collection: Integrating Health Care to Meet the Needs of the Mother–Infant Pair.

*Please see later in the article for the Editors' Summary*

The Maternal Health Task Force (MHTF) at Harvard School of Public Health and *PLOS Medicine* are pleased to announce the third year of our Open Access collection of research and commentary on maternal health. The continuation of this collaboration reflects our shared commitment to increasing the evidence base for approaches to improving maternal health and to making that evidence freely available for all. We are delighted to issue a call for papers for the third year of this partnership under the theme *Integrating Health Care to Meet the Needs of the Mother–Infant Pair*.

The Year 3 Collection builds on the successful first and second years of collaboration between the MHTF and PLOS. In the first year, the Quality of Maternal Health Care Collection included 18 original articles that examined topics such as the quality of care provided to women experiencing severe maternal morbidity in Ghana [1], the role of the “Midwives Service Scheme” in Nigeria [2], a prospective pilot study of the World Health Organization Safe Childbirth Checklist program [3], and a variety of other topics. The second year's Collection, Maternal Health Is Womens Health, to date comprises 30 original articles on a wide range of topics including socioeconomic disparities in maternity care for adolescents in India [4], the significance of minor ailments during pregnancy for women in Sri Lanka [5], and global estimates for syphilis in pregnancy and associated adverse outcomes [6].

The Year 1 and Year 2 Collections have helped build the evidence base for various approaches to improving the quality of maternal health care, and have placed maternal health in the context of women's health. This year, under the theme *Integrating Health Care to Meet the Needs of the Mother–Infant Pair*, we aim to strengthen the evidence for approaches to providing integrated care from pre-conception, throughout pregnancy, childbirth, and the postnatal period, to the child's early days and years of life. Through this Collection, we will contribute to a better understanding of how and when to integrate maternal and infant health care comprehensively, to include conditions such as HIV, malaria, exposure to environmental risks, and other situations that have a significant impact on both maternal and infant health.

## Year 3 Theme

Why the focus on integration and the continuum of care? We selected this year's theme for three main reasons.

### First, to Provide a Platform for Dissemination of New Evidence on Integration and the Continuum of Care

“Integration” and a focus on the “continuum of care” have become widely accepted approaches to program implementation in global health. While these approaches are conceptually sound, there is scant empirical evidence about the practical implementation of integrated programs and services for the mother–infant pair. Evidence from projects and programs, particularly in low-resource settings, will support the maternal and child health communities' efforts to adjust and scale up proven approaches.

### Second, to Offer a Venue for Information Sharing and Analyses of Conditions That Affect Mothers and Infants

This Collection will contribute to a more robust understanding of the toll that conditions such as malaria and HIV and AIDS take on mothers and infants, and how best to integrate care for maximum impact. Additionally, as treatment protocols for both malaria and HIV are rapidly evolving and changing, it is imperative that researchers closely examine emerging issues—and those of other conditions—to gather critical evidence to inform policy and service delivery and ensure that women and infants are provided with the highest-quality care possible.

### Third, to Discuss the Role of Integration in the Context of Universal Health Coverage (UHC) in the Post-2015 Development Agenda

As the world approaches the deadline for the Millennium Development Goals in 2015 and begins to consider what a post-2015 agenda might entail, a consensus is emerging among global health and development practitioners and policymakers that UHC is an essential means of achieving sustainable development. UHC involves integrated care for families, especially for mothers and their children. It is our hope that this year's Collection, with its focus on integration, will provide valuable evidence for the ongoing discussions about the post-2015 development agenda and the most effective approaches to meeting the needs of the mother–infant pair.

## Call for Papers

For the current MHTF–PLOS Collection on Maternal Health, we welcome primary research articles (using quantitative, qualitative, and mixed methods) and insightful commentary related to *Integrating Health Care to Meet the Needs of the Mother–Infant Pair*. Potential topics for articles might include the following:

1. **Robust analyses of health outcomes resulting from successful and unsuccessful efforts to offer integrated care.** We welcome, for example, results of independent evaluations of innovative models of integrated emergency care during delivery and the immediate neonatal period on newborn survival and maternal morbidity.
2. **Innovative approaches to identifying and addressing health needs along the continuum of care.** While packages of safe and effective interventions along the continuum of care have been identified, evidence on how to implement them in low-resource settings is limited. We welcome papers that describe, for example, the health system–related challenges that need to be overcome in order to equitably and successfully introduce packages that integrate reproductive, maternal, newborn, and child health care.
3. **Results and analyses of programs that comprehensively address maternal and newborn health care in the context of programs and services primarily focused on other conditions, such as HIV and AIDS, malaria, tuberculosis, and environmental health.**

Research articles should include robust evaluation of the impact of initiatives and interventions after their implementation including mother–infant outcomes related to health, quality of life, and quality of care. Commentary articles, targeted to the *PLOS Medicine* Essay, Policy Forum, or Health in Action sections, must be novel and well-argued. and include relevant evidence. Authors should refer to the *PLOS Medicine* Guidelines for Authors at http://www.plosmedicine.org/static/guidelines.action for specific submission requirements.

All papers should be submitted to *PLOS Medicine*, with a clear statement in the cover letter that they are intending to submit to the Maternal Health Task Force Collection. Authors for whom the publishing fee for research articles presents a barrier are encouraged to select the PLOS fee waiver when submitting an article. An initial decision will be made about papers' potential suitability for either *PLOS Medicine* or another PLOS journal. The authors will be informed of this decision, and papers considered appropriate for PLOS journals will then be peer-reviewed according to the specific journal's policies; no articles can be guaranteed acceptance at any journal. PLOS editors will retain all control over editorial decisions. If and when a paper is accepted for publication in a PLOS journal, it will be forwarded to the selection panel for the Collection. This panel, which is composed of PLOS and MHTF staff, will decide on articles' suitability for inclusion in the Collection.

We invite submissions now on the theme *Integrating Health Care to Meet the Needs of the Mother–Infant Pair*. Articles will stand the best chance of inclusion in the Year 3 Collection if they are submitted by 1^st^ April 2014.

PLOS and the MHTF look forward to our continued collaboration on this initiative.
